# Supplementary material for: Does eye-tracking have an effect on economic behavior?
Source: PLoS One. 2021 Aug 5;16(8):e0254867. doi: 10.1371/journal.pone.0254867 (PMC8341649; doi:10.1371/journal.pone.0254867)
Supplement: S1 Appendix — (PDF) [file pone.0254867.s001.pdf]

S1 Appendix. Abbreviated Instructions

This section presents a transcription of the abbreviated instructions for each game. The full script is available from the authors upon request.

General Instructions

Welcome and thank you for participating in our study. You will receive $10 for participating; this will be yours to keep. You will also have the opportunity to make more earnings based on your decisions, the decisions of the other players, and luck. So, please pay attention to the instructions.

Today you will play 4 games. At the end of the session, one of the games will be randomly selected for payment. The total amount you earn today will be paid to you in cash and privately at the end of the experiment. Please do not talk to other participants.

1. Dictator game

[*Page 1*] **Instructions**
In this game there are two players: Player 1 and Player 2. You will be randomly matched with another person in this room. You will not be told the identity of the person that you are matched with and the other person will not know your identity. One of you will be randomly assigned the role of Player 1 and the other the role of Player 2. Player 1 begins with 10 tokens and Player 2 begins with 0 tokens. There will be one period only.

In this game, 1 token is equal to $1.

Please raise your hand if you have any questions, otherwise please click NEXT to continue.

[*Page 2*]
Player 1's Decision:
Player 1 will be asked to decide to split 10 tokens between Player 1 and Player 2. Player 1 can choose any integer amount between 0 and 10 tokens.
Player 2's Decision:
Player 2 will be told the offer and has no choice to make.
Payoffs:
That is, if this game is selected for payment, Player 1's payoff (not including show-up fee) = 10 tokens minus the number of tokens transferred to Player 2. Player 2's payoff (not including show-up fee) = the amount Player 1 transferred.
Please raise your hand if you have any questions, otherwise please click NEXT to continue.

[*Page 3*] Once all decisions have been made the game will end. We will calculate payoffs based on the decisions made. Again, you will be randomly paid for one of the games at the conclusion of the experiment.

[*Page 4*] You are Player 1./You are Player 2.

[*Page 5. Decision screens*]
[*Page 6*] This concludes Game 1. Please wait for the experimenter to come.

2. Trust game

[*Page 1*] **Instructions**
In this game there are two players: Player 1 and Player 2. You will be randomly matched with another person in this room. You will not be told the identity of the person that you are matched with and the other person will not know your identity. One of you will be randomly assigned the role of Player 1 and the other the role of Player 2. Your payoffs will be determined by the decisions that you both make. Player 1 and

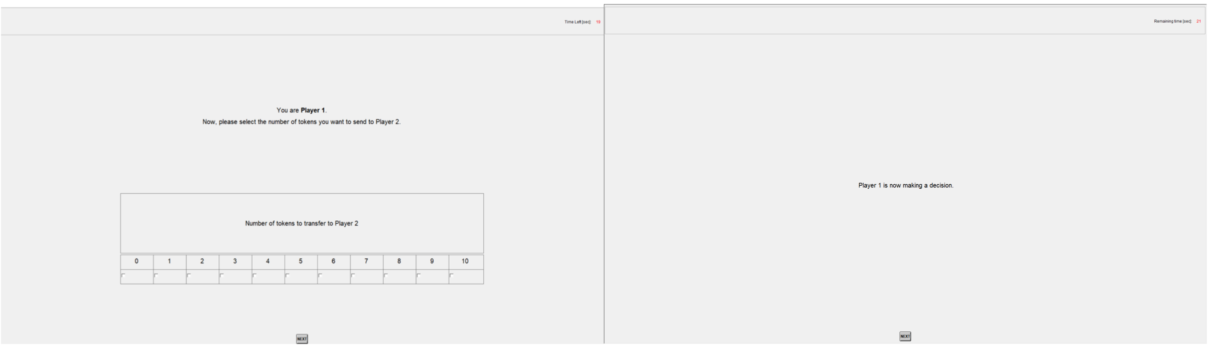

(a) Player 1 (b) Player 2

Figure 1: Dictator game

Player 2 both begin with 10 tokens.

In this game, 2 tokens are equal to $1.

Please raise your hand if you have any questions, otherwise please click NEXT to continue.

[*Page 2*] **Player 1's Decision:**
Player 1 moves first. Player 1 may send some, all, or none of the 10 tokens to Player 2. Each token sent to Player 2 will be tripled.For example, if Player 1 sends 2 tokens, Player 2 receives 6 tokens = (2 tokens × 3). If Player 1 sends 9 tokens, Player 2 receives 27 tokens = (9 tokens × 3).Player 2 will then decide how many tokens to send back to Player 1 and how many tokens to keep. Player 1 indicates how much to send to Player 2 by typing the appropriate amount of tokens on the decision screen.

[*Page 3*] **Player 2's Decision:**
Player 2 begins with 10 tokens. In addition, Player 2 receives three times the amount sent by Player 1. Player 2 may send back some, all, or none of the tripled amount to Player 1 (Player 2 keeps the 10 tokens they started with). Any tokens sent back to Player 1 will not be tripled. Once Player 1 has sent some, none, or all of their tokens, Player 2 will then decide how many tokens to send back to Player 1 and how many tokens to keep.

[*Page 4*] Once all decisions have been made the game will end. We will calculate payoffs based on the decisions made. Again, you will be randomly paid for one of the games at the conclusion of the experiment.

[*Page 5*] You are Player 1./You are Player 2.

[*Page 6*] *Decision screens*]

[*Page 7*] This concludes Game 2. Please wait for the experimenter to come.

3. Holt and Laury Risk Task

[*Page 1*] **Instructions**
In this game you will make ten decisions. Please make your choices carefully. The Decision Screen shows a choice between Option A and Option B. You will make one choice and record it in the choice column. Then you will move to the next Decision Screen. Please raise your hand if you have any questions, otherwise please click NEXT to continue.

[*Page 2*] Even though you will make ten decisions, if this game is selected for payment only one decision will affect your earnings. One of the decisions you make will be selected randomly for your payment. Since you do not know in advance which decision will be selected, your best approach is to make each decision as if it is the one that will be selected for payment. Each decision has an equal chance of being selected.

In this game, 1 token is equal to $4.

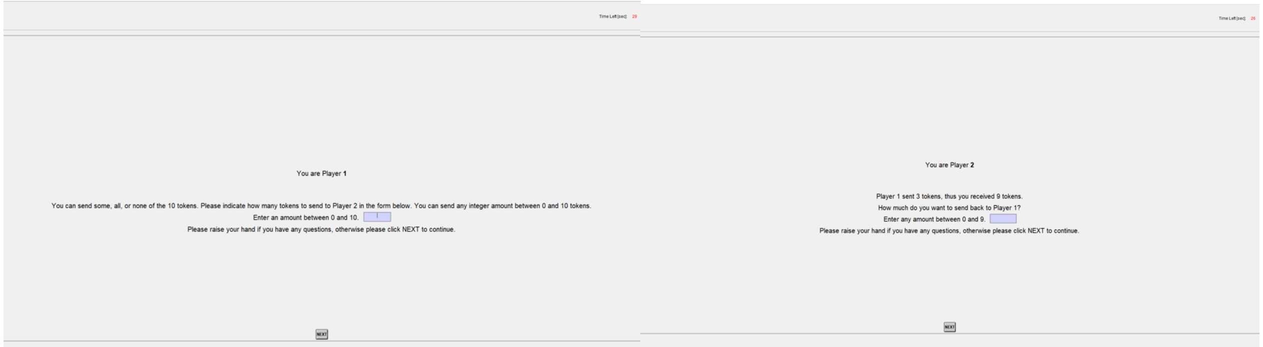

(a) Player 1

(b) Player 2

Figure 2: Trust game

Please raise your hand if you have any questions, otherwise please click NEXT to continue.

[*Page 3*] If this game is selected for payment, we will ask one of you to volunteer to draw a chip from a bag. The bag contains 10 chips numbered 1 through 10, one for each decision in this game. The chip will determine which decision will be the real decision. After pulling the chip, your payment will be determined based on your decision and a roll of a dice. This will be explained in the following screens.
Please raise your hand if you have any questions, otherwise please click NEXT to continue.

[*Page 4*] Once all decisions have been made the game will end. We will calculate payoffs based on the decisions made. Again, you will be randomly paid for one of the games at the conclusion of the experiment.

[*Page 5*] The following gives you an example of the decision screen. It reproduces the first decision. Option A pays either 2.00 toknes or 1.60 tokens. It depends on the roll of a ten-sided dice. The same is true for Option B, which yeilds either 3.85 tokens or 0.110 tokens depending on the roll of the dice.
Please raise your hand if you have any questions, otherwise please click NEXT to continue.

	Option A	Option B	Your Choice A or B	
Decision 1	2.00 tokens if Dice is 1<br>1.60 tokens if Dice is 2-10	3.85 tokens if Dice is 1<br>0.10 tokens if Dice is 2-10	A	B

[*Page 6. 10 Decisions were displayed in separate pages.*]
[*Page 7*] This concludes Game 3. Please wait for the experimenter to come.

4. Double Auction

[*Page 1*] **Instructions**
In this game there are two players: Seller and Buyer. You will be randomly matched with another person in this room. You will not be told the identity of the person that you are matched with and the other person will not know your identity. One of you will be randomly assigned the role of Seller and the other the role of Buyer. If this game is selected for payment, your payoffs will be determined by the decisions made by the Buyers and Sellers.

In this game, 1 token is equal to $1.

[*Page 2*] Every Seller can sell at most one unit of a fictitious good.The minimum assigned price at which the Seller can sell the unit of this good in any period will appear on the top left corner of the screen. The Buyer can buy at most one unit of the fictitious good. The maximum assigned price at which the Buyer can buy a unit of this good will appear on the top left corner of the screen.
Your assigned value will be private information. There will be 10 trading periods of 2 minutes each. Your

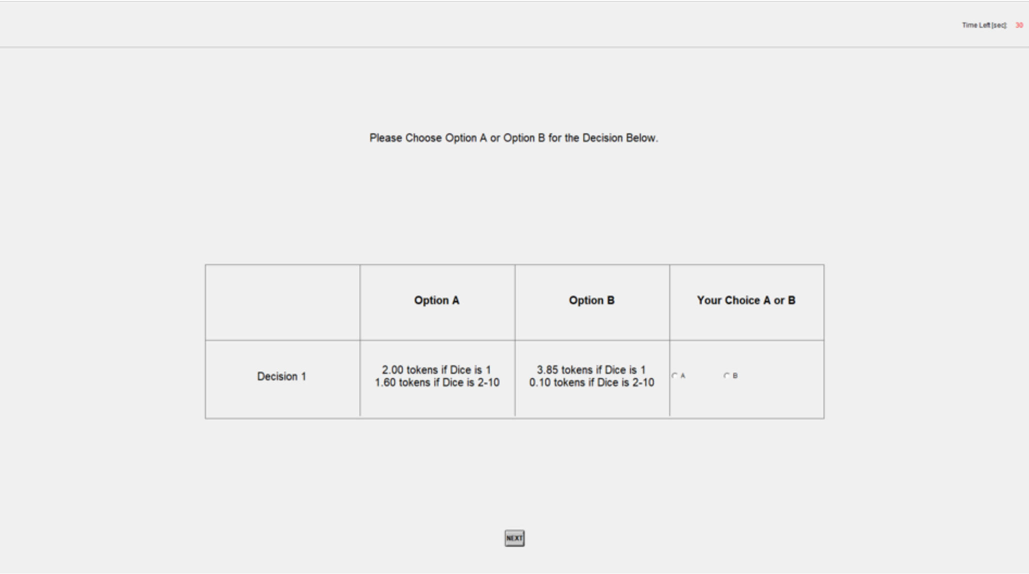

Figure 3: Holt and Laury

assigned price will remain the same for all ten periods.
Please raise your hand if you have any questions, otherwise please click NEXT to continue.

[*Page 3*] Before the game starts, the computer will randomly assign half of the participants the role of Buyers and the other half of the participants the role of Sellers. At the beginning of each trading period, two pieces of information will appear on the top left corner of your screen: your role (either Buyer or Seller) and your assigned price for the good. Please remember that your role and assigned price will remain the same for all ten trading periods. The first trading period will start when every player is done reading the instructions.

[*Page 4*] The goal of every trader is to maximize their payoff at every trading period. Thus, each Seller has to try to sell the good at the highest possible price, and each Buyer has to try to buy the good at the lowest possible price. Prices must be multiples of 0.5.
Please raise your hand if you have any questions, otherwise please click NEXT to continue.

[*Page 5*] **Important remarks:**
Buyers cannot bid above their assigned maximum prices. That is, Buyers are only allowed to propose prices below or equal to their assigned prices for the good. If Buyers do not make a purchase, Buyers do not earn anything in the period. Similarly, Sellers cannot ask values below their assigned minimum prices. That is, Sellers are only allowed to propose prices above or equal to their assigned prices for the good. If Sellers do not make a sale, Sellers do not earn anything or incur any cost in that period.

[*Page 6*] A transaction is finalized when a Buyer accepts a Seller's offer, or when a Seller accepts a Buyer's bid. The Buyer and Seller making the deal are to drop out of the market, making no more bids, offers, or contracts for the remainder of that trading period. This process continues for up to two minutes depending on the volume of trading.If Buyers or Sellers resubmit bids or offers, a transaction will automatically cancel all prior bids and offers made by the Buyers and Sellers involved. Bids and offers made by those who are not involved in the transaction do not have to be reentered. The highest bid and the lowest offer will be displayed at all times on your screen.

[*Page 7*] Your payoff will be equal to:
-the difference between the closing price and your assigned minimum price if you are a Seller;
-the difference between your assigned maximum price and the closing price if you are a Buyer.

Once you complete a transaction, your payoff for that period will appear on the screen.

[*Page 8*] Once all decisions have been made the game will end. We will calculate payoffs based on the decisions made. Again, you will be randomly paid for one of the games at the conclusion of the experiment.

[*Page 9. 10 Periods of auctions were conducted.*]
Period: 1
You are a: **SELLER**
Price of Good: [ ]
Trading will start soon!

[*Page 10. 10 Periods of auctions were conducted.*]

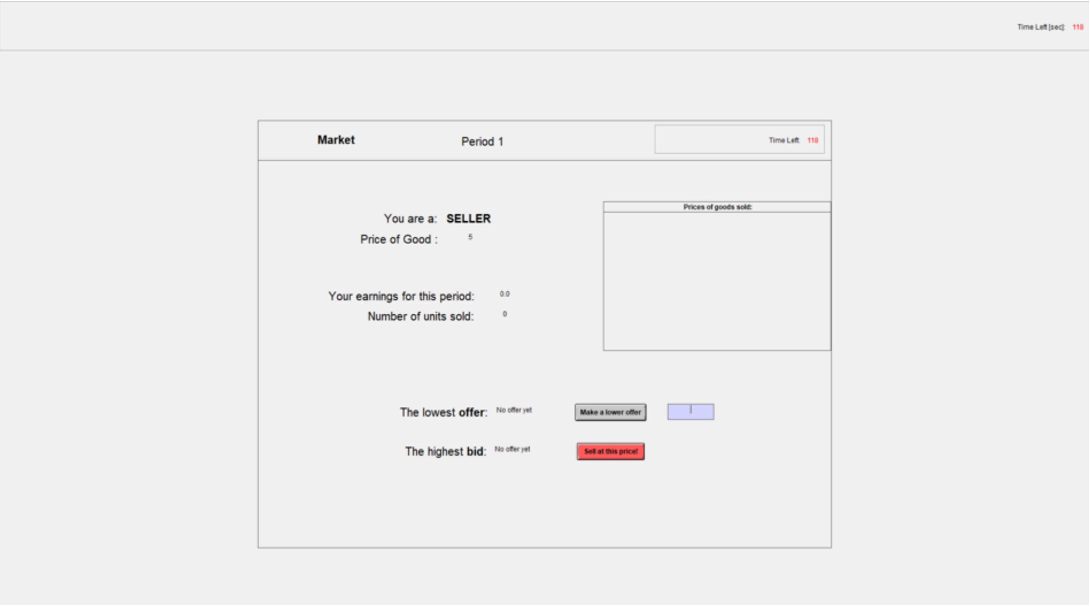

Figure 4: Double Auction

[*Page 10*] This concludes Game 4.

5. Eckel and Grossman Gambling Task

[*Page 1*] **Instructions**
In this game, you will be selecting from one of six available gambles. The six different gambles will be listed on your GAMBLE SELECTION screen. You must choose one, and only one, of these gambles. To select a gamble, type the option that corresponds to that gamble into the form on the Decision Screen. Each gamble has two possible outcomes, High Amount or Low Amount, with equal probability of the event occurring. If this game is selected for payment, your compensation for the study will be determined by:
1) which of the six gambles you select; and
2) which of the two possible events occur.

[*Page 2*] Remember, every gamble has two possible outcomes that can occur with equal chance. At the end of this session, if this game is selected for payment, we will ask one of you to volunteer to draw a chip from a bag. The bag will contain two chips. One chip has an H on it representing High Amount. The other chip has an L on it representing Low Amount. If the volunteer pulls out the H chip, you earn the High Amount from the choice you picked. If the volunteer pulls out the L chip, then you earn the Low Amount from the

choice you picked. Because there are only two chips, each chip has equal chance to be pulled out of the bag.

In this game, 2 tokens are equal to $1.

Please raise your hand if you have any questions, otherwise please click NEXT to continue.

[*Page 3*] Once all decisions have been made the game will end. We will calculate payoffs based on the decisions made. Again, you will be randomly paid for one of the games at the conclusion of the experiment.

[*Page 4. Decision screen*]

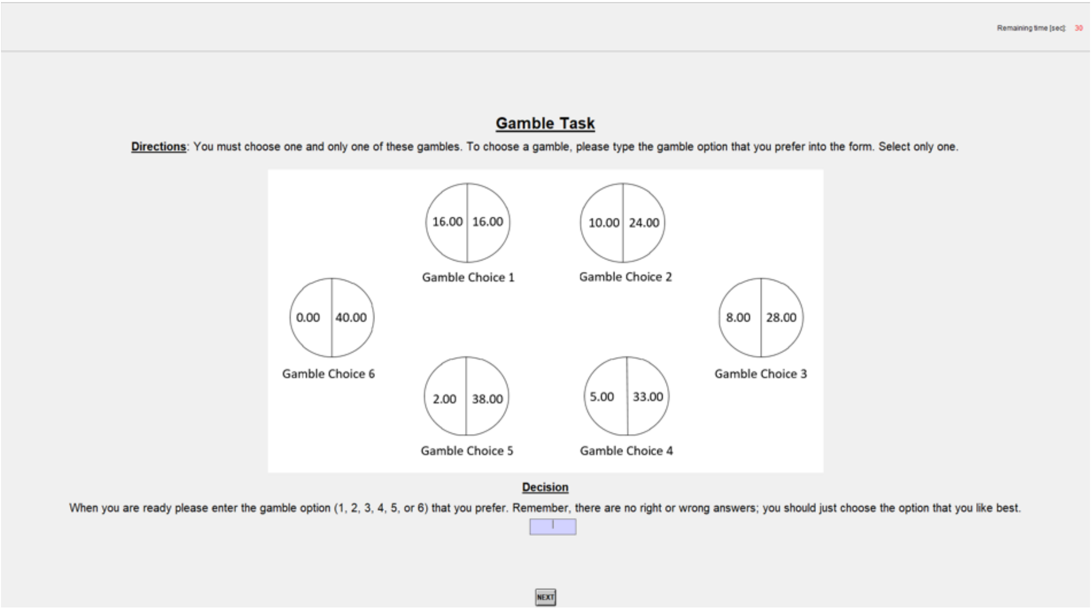

Figure 5: Eckel and Grossman

[*Page 5*] This concludes Game 1. Please wait for the experimenter to come.

6. Public Goods game

[*Page 1*] **Instructions**

In this game, you will participate in a total of 12 periods (2 practice and 10 real). In each period, you will be randomly assigned to a group of 4 members. Each member will be endowed with 100 tokens and must decide how to divide the tokens between two accounts:

1) Private Account

2) Public Account

In this game, 1 token is equal to 10 cents ($0.10).

[*Page 2*] The composition of your group will change every period. Each period, you will be randomly reassigned to a new group of 4 members. At no point in the experiment will the identities of the other group members be revealed to you, nor will your identity be revealed to them. In other words, the group members will remain anonymous to one another. You will be endowed with 100 tokens in every period and must decide how many tokens to invest in the private account and how many tokens to invest in the public account. Information about the two accounts is presented in the next four screens.

[*Page 3*] Private Account: Every token you invest in the private account will yield you a return of 10 cents. The other members in your group will not be affected by your investment in the private account. Here are a few examples to illustrate:
Example 1: Suppose you invest 100 tokens in the private account. Then you will get 1,000 cents (or $10.00) from this account and the other members of your group will not be affected for that period.
Example 2: Suppose you invest 50 tokens in the private account. Then you will get 500 cents (or $5.00) from this account and the other members of your group will not be affected for that period.
Example 3: Suppose you invest 0 tokens in the private account. Then you will get 0 return from this account and the other members of your group will not be affected for that period.
Please raise your hand if you have any questions, otherwise please click NEXT to continue.

[*Page 4*] Public Account: Every token you invest in the public account will yield a return of half a cent to each member of your group. Also, every token that any of your group members invests in the public account will yield a return of half a cent to each member of your group.
This means that your return from the public account will depend on the total number of tokens that you and the other members of your group invest in this account. The more the group invests in the public account, the greater the return to each member of the group from this account.
Here are a few examples to illustrate:
Example 1: Suppose you invest 0 tokens in the public account and the other three members of your group invest a total of 200 tokens in the public account. Then, the total number of tokens invested by your group in the public account is 200 which means that every member of your group earns 200 $\times$ 0.5 = 100 tokens $\times$ 10 cents = 1,000 cents (or $10.00) from the public account for that period.
Please raise your hand if you have any questions, otherwise please click NEXT to continue.

[*Page 5*] **Important remarks:**
Example 2: Suppose you invest 100 tokens in the public account and the other three members of your group invest a total of 0 tokens in the public account. Then the total number of tokens invested by your group in the public account is 100 which means that every member of your group earns 100 $\times$ 0.5 = 50 tokens $\times$ 10 cents = 500 cents (or $5.00) from the public account for that period.
Example 3: Suppose you invest 100 tokens in the public account and the other three members of your group invest a total of 300 tokens in the public account. Then the total number of tokens invested by your group in the public account is 400 which means that every member of your group earns 400 $\times$ 0.5 = 200 tokens $\times$ 10 cents = 2,000 (or $20.00) from the public account for that period.
Please raise your hand if you have any questions, otherwise please click NEXT to continue.

[*Page 6*] Your decisions and earnings in every period are confidential. This means that you will not be given information about the investment decisions or earnings of any of your group members, nor will they be given information about your investment decisions or earnings. So you must make your decision without knowing what the other members in your group are deciding.
After each period, the only information you will be given is:
1) Number of tokens you invested in the private and public accounts
2) The total number of tokens invested by your group (including you) in the public account
3) Your earnings for that period
At the end of the session, if this game is selected for payment, 1 of the 10 real periods will be randomly selected as binding. We will ask one of you to volunteer to draw a chip from a bag. The bag will contain ten chips, one for each period in this game.
Please raise your hand if you have any questions, otherwise please click NEXT to continue.

[*Page 6*] Once all decisions have been made the game will end. We will calculate payoffs based on the decisions made. Again, you will be randomly paid for one of the games at the conclusion of the experiment.

[*Page 7. 2 practice rounds were conducted.*]
Practice Round 1
Please enter your contribution to the private and public accounts and click NEXT. Remember, that your

combined contribution to both the private account and public account must equal 100 tokens.
Group 1
Your endowment is 100 tokens
Your contribution (tokens) in the private account:
Your contribution (tokens) in the public account:

[*Page 8. 2 practice rounds were conducted.*]
You are in Group 1
Your Profit

Your contribution (tokens) in your private account:	
Your contribution (tokens) into the public account:	
Total group investment (tokens) into the public account	
Your earnings for this period	$

[*Page 9-10. 10 periods of public goods game were conducted.*]

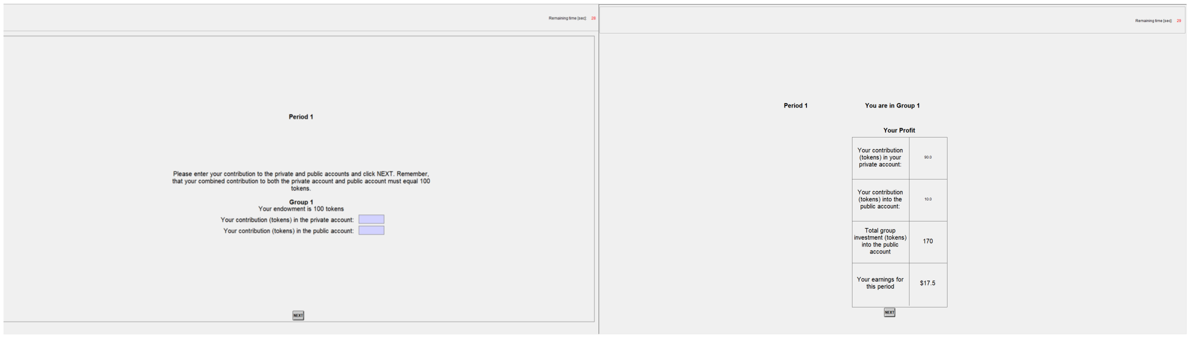

(a) Page 9. Decision screen (b) Page 10. Corresponding profits

Figure 6: Public Goods game

[*Page 11*] This concludes Game 2. Please wait for the experimenter to come.

7. Ultimatum game

[*Page 1*] **Instructions**
In this game there are two players: Player 1 and Player 2. You will be randomly matched with another person in this room. You will not be told the identity of the person that you are matched with and the other person will not know your identity. One of you will be randomly assigned the role of Player 1 and the other the role of Player 2. Player 1 begins with 10 tokens and Player 2 begins with 0 tokens. There will be one period only.

In this game, 1 token is equal to $1.

Please raise your hand if you have any questions, otherwise please click NEXT to continue.

[*Page 2*]
Player 1's Decision:
Player 1 will be asked to propose to split 10 tokens between him/herself and Player 2. Player 1 can choose any integer amount between 0 and 10 tokens.
Player 2's Decision:
Player 2 will be told the offer made by Player 1 and asked to choose to either **accept** or **reject** the offer. If Player 2 **accepts** the offer, both Player 1 and Player 2 receive the amounts specified in the **accepted** offer. If Player 2 **rejects** the offer, both players receive zero earnings for this game.
Please raise your hand if you have any questions, otherwise please click NEXT to continue.

[*Page 3*] Once all decisions have been made the game will end. We will calculate payoffs based on the decisions made. Again, you will be randomly paid for one of the games at the conclusion of the experiment.

[*Page 4*] You are Player 1./You are Player 2.

[*Page 5. Decision screens*]

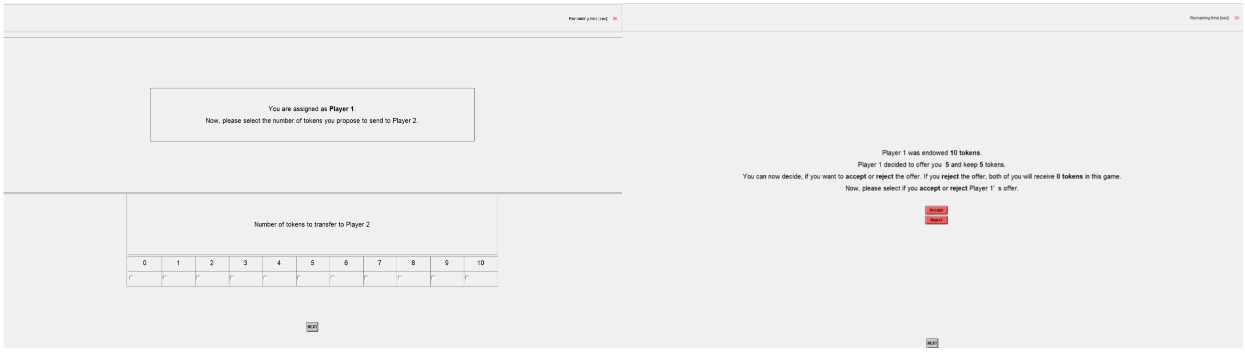

(a) Player 1 (b) Player 2

Figure 7: Ultimatum game

[*Page 6*] This concludes Game 3. Please wait for the experimenter to come.

8. Cheating game

[*Page 1*] **Instructions**
In this game, you will see 5 numbers on the screen. You will determine your own payoff by recording the first number that you see. Then, you will move to the next period. You will play this game for 10 periods.

In this game, 1 token is equal to $1.

In other words, if you report seeing "0" first, you receive 0 tokens. If you report seeing "2" first, you receive 2 tokens, or if you report seeing "4" first, you receive 4 tokens.

[*Page 2*] Each screen will present to you 5 numbers. The numbers are 0, 2, 4, 6, and 8 in different orders. Your task is to report the first number that you see and record the number on the next screen. The position of the numbers on the screen are determined by a computer generated output. Before this session began, we called a participant to monitor the randomization process.
Please raise your hand if you have any questions, otherwise please click NEXT to continue.

[*Page 3*] At the end of this session, if this game is selected for payment, we will ask one of you to volunteer to draw a chip from a bag. The bag will contain ten chips, one for each of the periods in this game. Each period has an equal chance of being selected.
Once all decisions have been made the game will end. We will calculate payoffs based on the decisions made. Again, you will be randomly paid for one of the games at the conclusion of the experiment.

[*Page 4-5. 10 periods of cheating games were conducted.*]

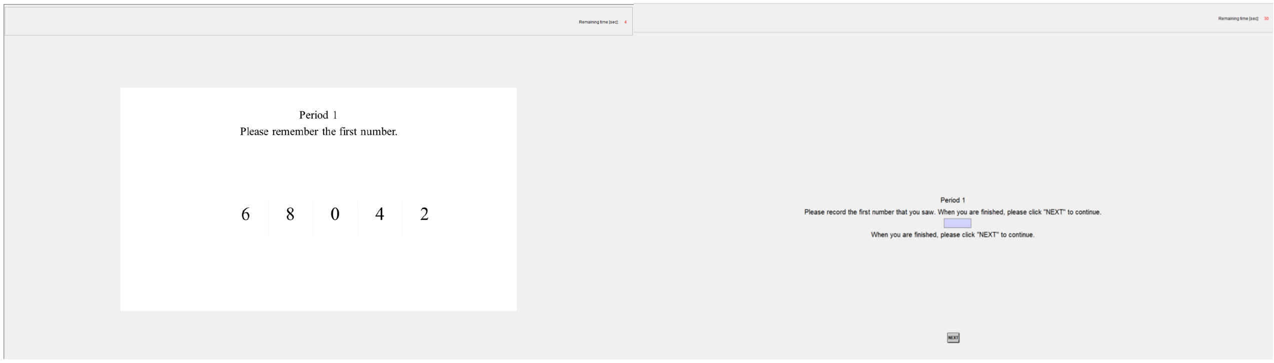

(a) Page 4. Decision screen (b) Page 5. Decision screen

Figure 8: Cheating game

[*Page 6*] This concludes Game 4.
