# Supplementary material for: Does eye-tracking have an effect on economic behavior?
Source: PLoS One. 2021 Aug 5;16(8):e0254867. doi: 10.1371/journal.pone.0254867 (PMC8341649; doi:10.1371/journal.pone.0254867)
Supplement: S2 Appendix — (PDF) [file pone.0254867.s002.pdf]

S2 Appendix. Figures for half of the game

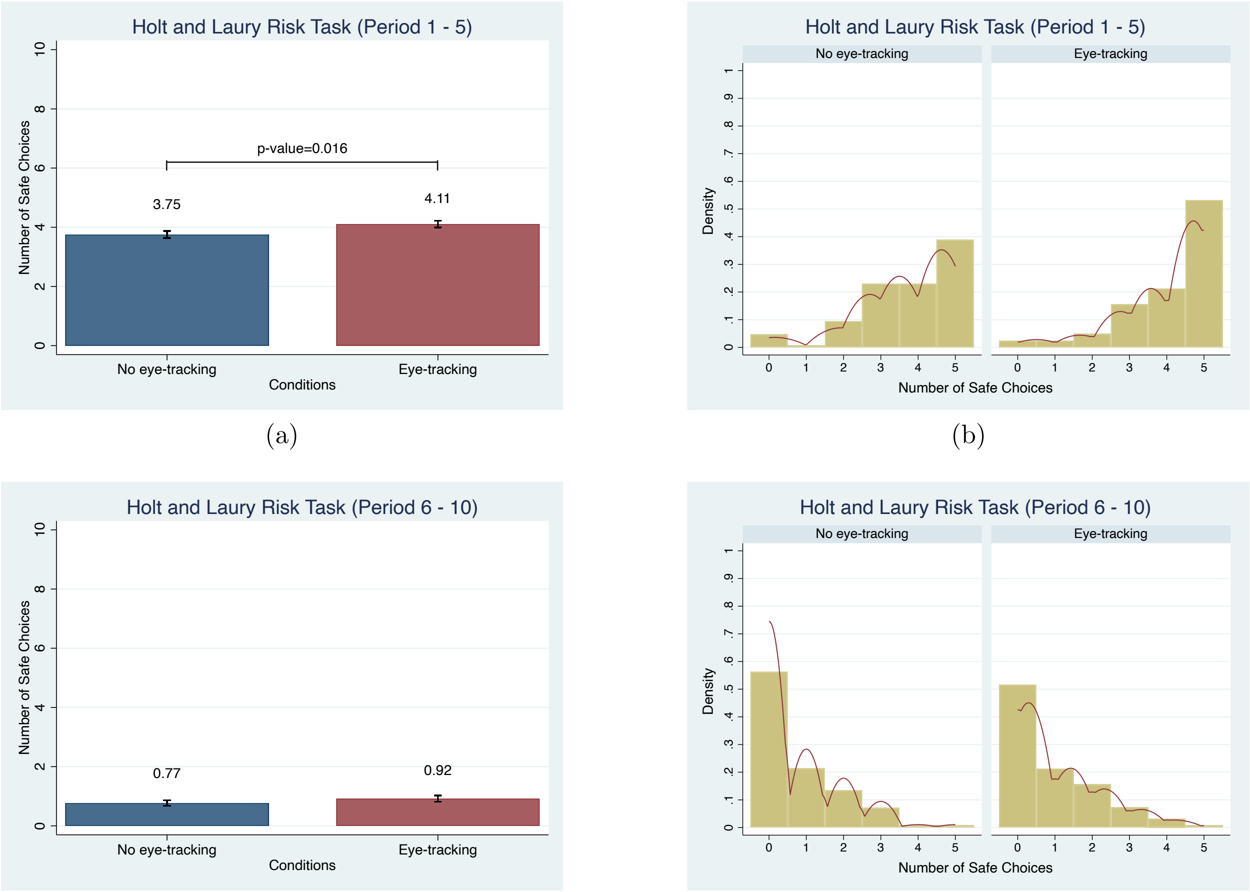

(a) (b) (c) (d)

Figure B1: Mean and distribution comparisons in Holt and Laury.

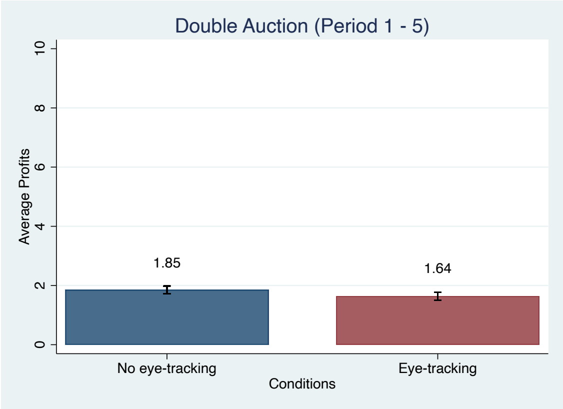

(a)

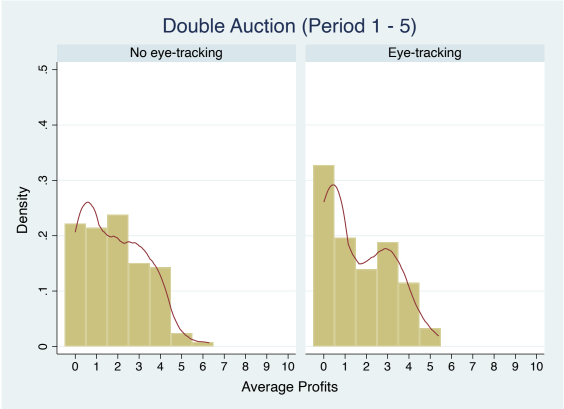

(b)

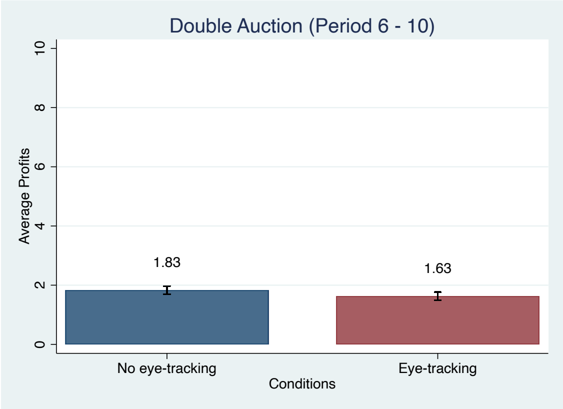

(c)

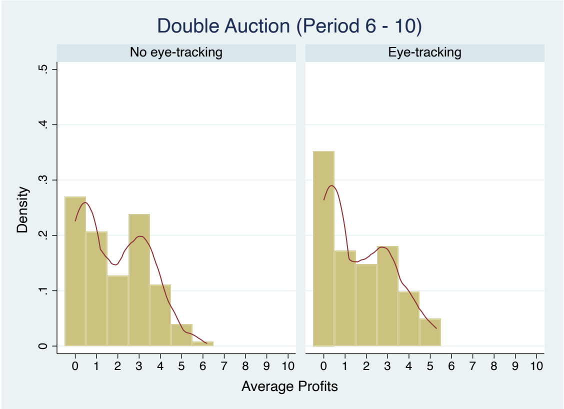

(d)

Figure B2: Mean and distribution comparisons in Double Auction.

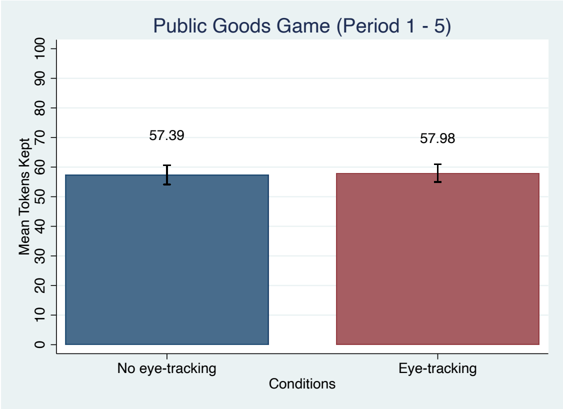

(a)

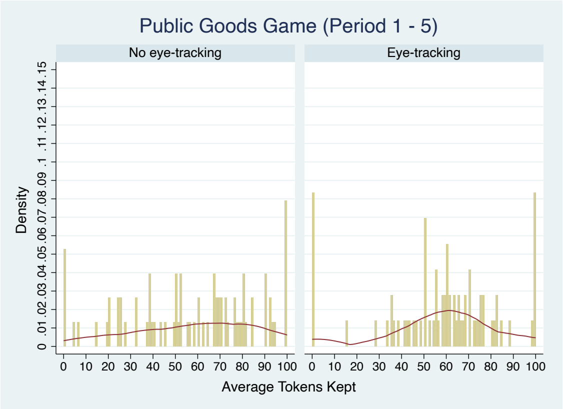

(b)

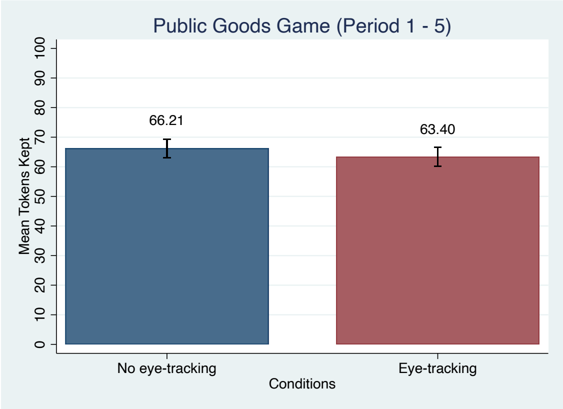

(c)

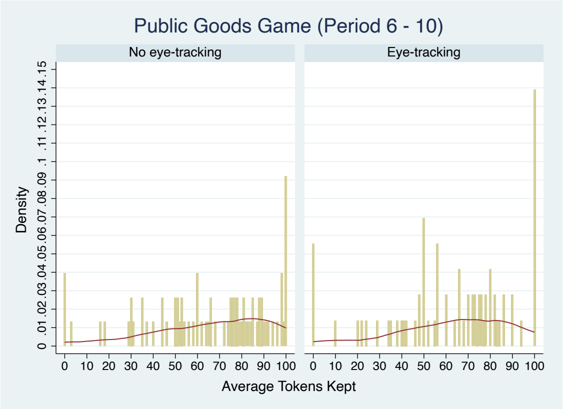

(d)

Figure B3: Mean and distribution comparisons in Public Goods game.

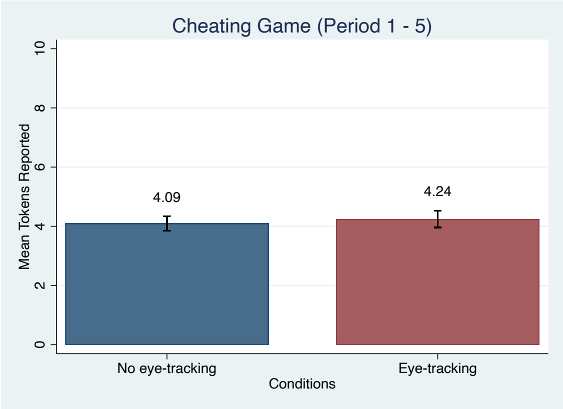

(a)

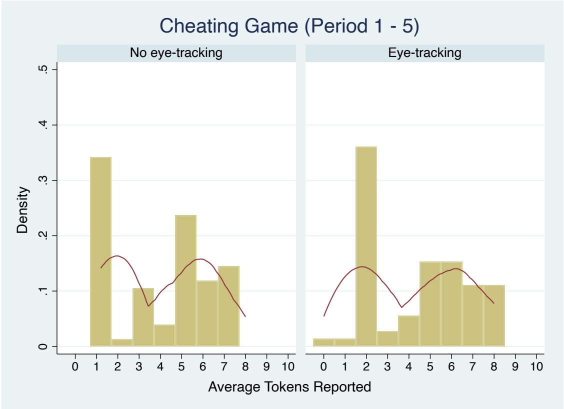

(b)

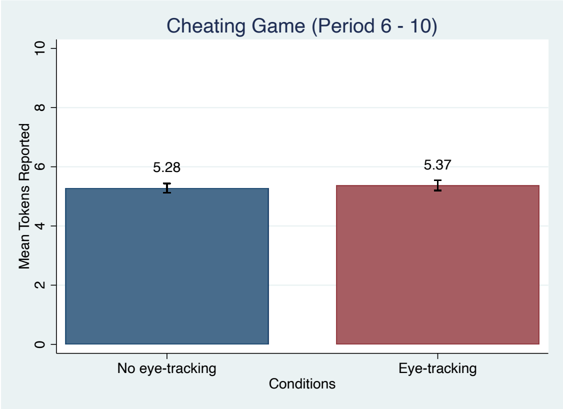

(c)

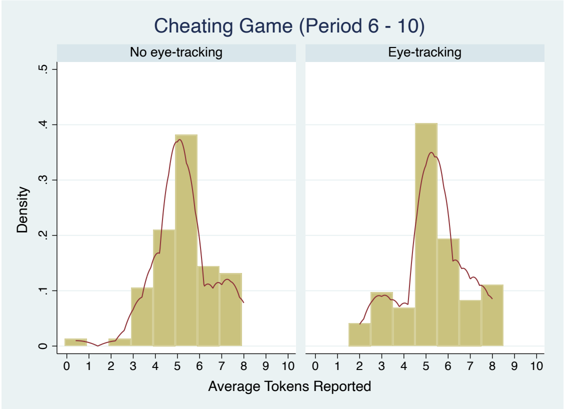

(d)

Figure B4: Mean and distribution comparisons in Cheating game.
