# Supplementary material for: Does eye-tracking have an effect on economic behavior?
Source: PLoS One. 2021 Aug 5;16(8):e0254867. doi: 10.1371/journal.pone.0254867 (PMC8341649; doi:10.1371/journal.pone.0254867)
Supplement: S3 Appendix — (PDF) [file pone.0254867.s003.pdf]

S3 Appendix. Additional figures for Double Auction

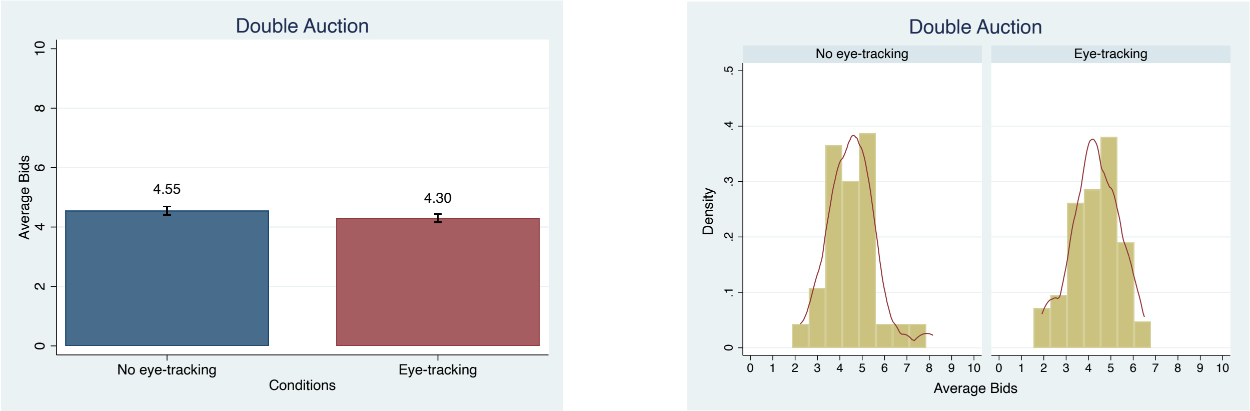

(a) (b)

Figure C1: Mean and distribution comparisons in bids.

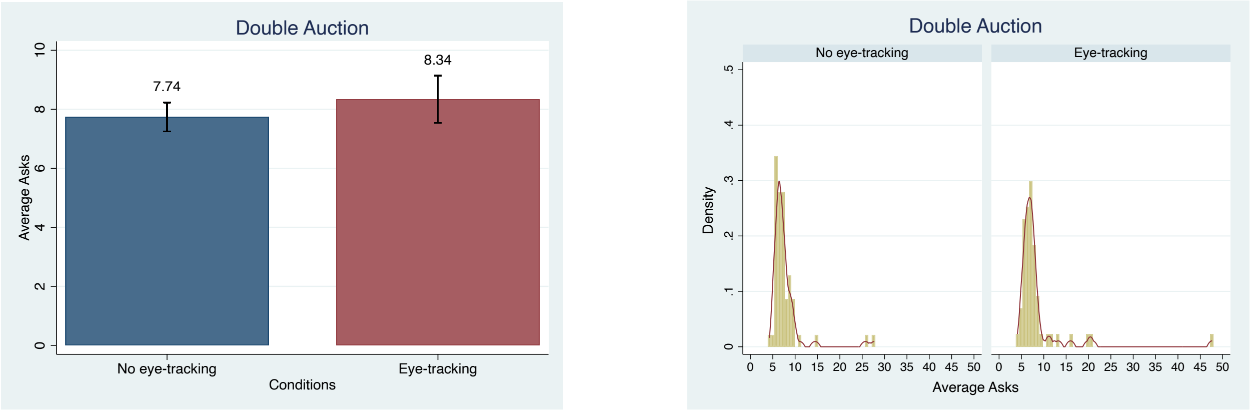

(a) (b)

Figure C2: Mean and distribution comparisons in asks.

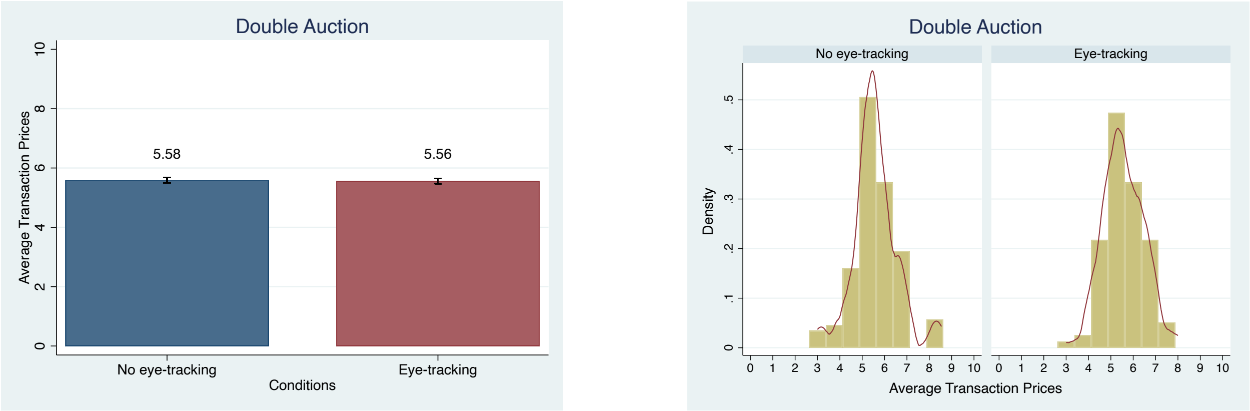

(a) (b)

Figure C3: Mean and distribution comparisons in transaction prices.

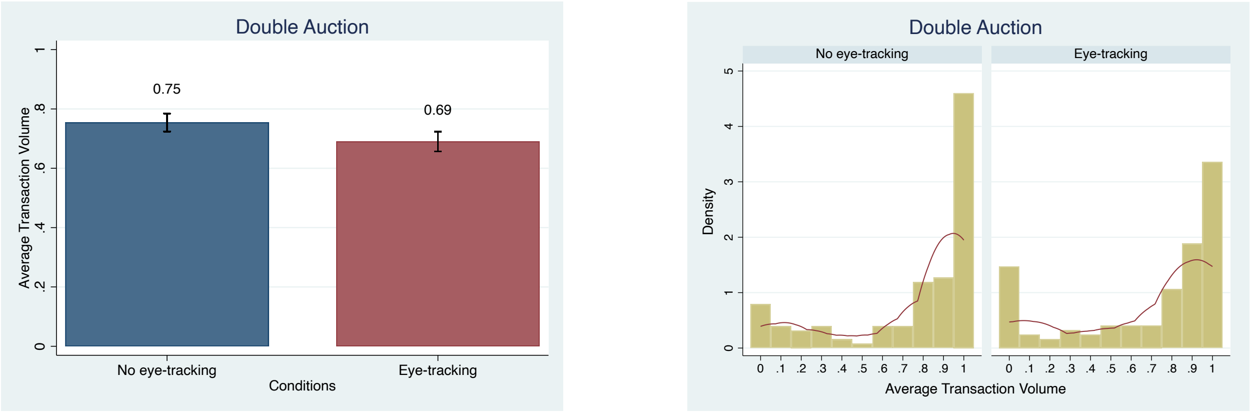

(a) (b)

Figure C4: Mean and distribution comparisons in transaction volume.
