# Supplementary material for: Does eye-tracking have an effect on economic behavior?
Source: PLoS One. 2021 Aug 5;16(8):e0254867. doi: 10.1371/journal.pone.0254867 (PMC8341649; doi:10.1371/journal.pone.0254867)
Supplement: S4 Appendix — (PDF) [file pone.0254867.s004.pdf]

# S4 Appendix. Additional tables

Table D1: Probit estimates for Player 2 in Ultimatum game.

| | (1) | (2) | (3) | (4) |
|---|---|---|---|---|
| Eye-tracking | -0.036 | -0.275 | -0.410 | -0.717 |
| | (0.355) | (0.436) | (0.314) | (0.446) |
| Male | | -0.373 | | 0.130 |
| | | (0.382) | | (0.561) |
| Age | | -0.086** | | -0.041 |
| | | (0.035) | | (0.040) |
| Sophomore | | | | |
| | | | | |
| Junior | | -0.315 | | -0.924 |
| | | (1.116) | | (1.009) |
| Senior + | | -0.151 | | -0.186 |
| | | (0.730) | | (0.660) |
| Master | | 0.638 | | 0.022 |
| | | (0.742) | | (0.714) |
| Ph.D. | | 0.622 | | -0.446 |
| | | (0.615) | | (0.710) |
| African American | | | | |
| | | | | |
| Asian | | 0.564 | | 0.349 |
| | | (0.550) | | (0.649) |
| Others | | | | |
| | | | | |
| $45,000-$49,000 | | -1.214 | | -0.985 |
| | | (0.803) | | (0.954) |
| $50,000-$59,000 | | | | |
| | | | | |
| >$60,000 | | 0.682 | | 0.566 |
| | | (0.424) | | (0.562) |
| Initial offer | | | 0.465** | 0.479** |
| | | | (0.197) | (0.211) |
| Constant | 1.003*** | 2.486** | -0.737 | 0.119 |
| | (0.287) | (1.041) | (0.824) | (1.642) |
| N | 74 | 59 | 74 | 59 |
| Pseudo R-sq. | 0.000 | 0.163 | 0.194 | 0.300 |

Note. Coefficients from probit estimation are reported. Standard errors are presented in parentheses and clustered at the session level. The dependent variable is the acceptance rate. In columns 2 and 4, 3 observations for sophomore, 1 observation for Black, 7 observations for Others, and 3 observations for $50,000-$59,000 were dropped because these observations predict success perfectly and 1 observation with missing gender was dropped. Senior$^{+}$ includes both senior and 5th year in undergraduate. * Statistically significant at 10% level; ** at 5% level; *** at 1% level.

## Table D2: Results from previous studies.

| Games | | Literature |
|---|---|---|
| Dictator Game | Player 1 sent average of 28 percent of the endowment. | [1] |
| Trust Game | Player 1 sent average of 70 percent of the endowment was sent, and 45 percent of Player 2s returned at least 10 tokens. | [2] |
| | Player 1 sent 50 percent of the endowment, and Player 2 returned 37 percent of the available funds they could return. | [3] |
| Holt and Laury | Number of safe choices was 5.2. | [4] |
| | Number of safe choices was 6.3. Subjects were Texas A&M University students. | [5] |
| Double Auction | Average profits was $0.88 in 15 rounds. | [6] |
| Eckel and Grossman | Gamble choice 3 was most chosen. Subjects were Texas A&M University students. | [5] |
| | Gamble choice 3 was most chosen. | [7] |
| Public Goods game | Average of 62.3 percent of the endowment was kept. | [8] |
| | Average of 33 percent of the endowment was kept. | [9] |
| Ultimatum Game | Player 1 sent 40 percent of the endowment, and 16 percent of the offers were rejected by Player 2. | [10] |
| Cheating Game | Average number of reported tokens was 4.11. | [11] |

## Table D3: Poisson regression estimates for Holt and Laury and Eckel and Grossman tasks.

| | (1)<br>Holt and Laury | (2)<br>Holt and Laury | (3)<br>Eckel and Grossman | (4)<br>Eckel and Grossman |
|---|---|---|---|---|
| Eye-tracking | 0.105** | 0.114** | 0.036 | 0.032 |
| | (0.047) | (0.048) | (0.060) | (0.054) |
| Male | | -0.147** | | -0.078 |
| | | (0.061) | | (0.058) |
| Age | | 0.020** | | -0.016 |
| | | (0.009) | | (0.010) |
| Sophomore | | -0.134 | | -0.054 |
| | | (0.088) | | (0.064) |
| Junior | | -0.115 | | -0.187 |
| | | (0.094) | | (0.128) |
| Senior$^{+}$ | | -0.137* | | -0.148*** |
| | | (0.081) | | (0.054) |
| Master | | -0.172* | | -0.046 |
| | | (0.104) | | (0.080) |
| Ph.D. | | -0.190 | | 0.072 |
| | | (0.132) | | (0.143) |
| African American | | -0.126 | | -0.394* |
| | | (0.154) | | (0.210) |
| Asian | | -0.038 | | 0.075 |
| | | (0.073) | | (0.058) |
| Others | | -0.081 | | -0.113 |
| | | (0.114) | | (0.074) |
| $45,000-$49,000 | | -0.095 | | -0.171 |
| | | (0.119) | | (0.164) |
| $50,000-$59,000 | | 0.049 | | 0.061 |
| | | (0.068) | | (0.118) |
| >$60,000 | | 0.029 | | 0.030 |
| | | (0.074) | | (0.056) |
| Constant | 1.508*** | 1.272*** | 1.480*** | 1.930*** |
| | (0.028) | (0.184) | (0.034) | (0.182) |
| N | 248 | 246 | 148 | 144 |
| Pseudo R-sq. | 0.003 | 0.016 | 0.000 | 0.020 |

Note. Coefficients from Poisson estimation are reported. Standard errors are presented in parentheses and clustered at the session level. The dependent variables are the number of safe choices. Senior$^{+}$ includes both senior and 5th year in undergraduate. 2 observations were dropped for column 2 and 4 subjects were dropped in column 4 because of missing demographic information. * Statistically significant at 10% level; ** at 5% level; *** at 1% level.

Table D4: Regression estimates for Double Auction and Public Goods game controlling for time trend.

| | (1) | (2) | (3) | (4) |
|---|---|---|---|---|
| | Double Auction | | Public Goods | |
| Eye-tracking | -0.206 | -0.165 | -1.110 | 3.283 |
| | (0.187) | (0.199) | (4.141) | (3.992) |
| Time trend | 0.001 | 0.002 | 1.382*** | 1.397*** |
| | (0.008) | (0.008) | (0.267) | (0.275) |
| Male | | -0.137 | | -12.515*** |
| | | (0.196) | | (4.205) |
| Age | | -0.053 | | 1.347** |
| | | (0.037) | | (0.561) |
| Sophomore | | 0.518 | | 5.079 |
| | | (0.344) | | (8.594) |
| Junior | | 0.326 | | -4.562 |
| | | (0.322) | | (9.207) |
| Senior$^{+}$ | | 0.251 | | -3.098 |
| | | (0.315) | | (6.900) |
| Master | | 0.568 | | -7.937 |
| | | (0.385) | | (8.066) |
| Ph.D. | | 1.053* | | -19.318* |
| | | (0.591) | | (10.040) |
| Black | | -1.029*** | | 4.158 |
| | | (0.318) | | (9.912) |
| Asian | | 0.023 | | 8.736 |
| | | (0.245) | | (5.424) |
| Others | | -0.236 | | 0.221 |
| | | (0.301) | | (7.000) |
| $45,000-$49,000 | | -0.204 | | -2.436 |
| | | (0.388) | | (5.109) |
| $50,000-$59,000 | | 0.143 | | 4.850 |
| | | (0.363) | | (7.766) |
| >$60,000 | | -0.190 | | -8.978 |
| | | (0.239) | | (5.706) |
| Constant | 1.836*** | 2.795*** | 54.196*** | 30.808** |
| | (0.133) | (0.774) | (3.412) | (12.281) |
| N | 2480 | 2460 | 1480 | 1440 |
| R-squared | 0.004 | 0.047 | 0.014 | 0.102 |

Note. Coefficients from OLS estimations are reported. Standard errors are presented in parentheses and clustered at the individual level. The dependent variables used in each game are: the average profits for 10 periods in Double Auction; the average number of tokens kept in Public Goods game. Senior$^{+}$ includes both senior and 5th year in undergraduate. 20 observations (2 subjects with 10 periods) in column 2 were dropped because 2 subjects did not report income level while 40 observations (4 subjects with 10 periods) in column 4 were dropped because 4 subjects did not report gender or income level. The results for bids, asks, transaction volume, and transaction price are similar and are available from the authors upon request. * Statistically significant at 10% level; ** at 5% level; *** at 1% level.

## Table D5: Cumulative number of calibration attempts in each game.

| Number of Calibration Attempts | Dictator game | Trust game (Player 1) | Trust game (Player 2) | Holt and Laury | Double Auction Profits | Eckel and Grossman | Public Goods | Ultimatum game (Player 1) | Ultimatum game (Player 2) | Cheating game |
|---|---|---|---|---|---|---|---|---|---|---|
| 1 | 66 | 0 | 0 | 12 | 0 | 36 | 0 | 0 | 0 | 0 |
| 2 | 8 | 29 | 27 | 8 | 11 | 21 | 30 | 0 | 0 | 0 |
| 3 | 10 | 2 | 5 | 51 | 6 | 5 | 18 | 13 | 12 | 0 |
| 4 | 4 | 7 | 9 | 15 | 49 | 3 | 11 | 10 | 8 | 17 |
| 5 | 3 | 0 | 4 | 14 | 13 | 5 | 6 | 7 | 7 | 22 |
| 6 | 1 | 4 | 0 | 9 | 18 | 1 | 4 | 3 | 3 | 12 |
| 7 | 0 | 3 | 1 | 3 | 9 | 0 | 2 | 1 | 1 | 8 |
| 8 | 0 | 1 | 0 | 5 | 2 | 1 | 0 | 2 | 3 | 5 |
| 9 | 0 | 0 | 0 | 4 | 6 | 0 | 0 | 0 | 1 | 5 |
| 10 | 0 | 0 | 0 | 1 | 4 | 0 | 0 | 0 | 0 | 1 |
| 11 | 0 | 0 | 0 | 0 | 2 | 0 | 1 | 0 | 0 | 1 |
| 12 | 0 | 0 | 0 | 0 | 1 | 0 | 0 | 0 | 1 | 0 |
| 13 | 0 | 0 | 0 | 0 | 1 | 0 | 0 | 0 | 0 | 1 |
| N | 92 | 46 | 46 | 122 | 122 | 72 | 72 | 36 | 36 | 72 |

Note. Frequency of the cumulative number of calibration attempts in each game is reported.



## Table D6: Regression estimates controlling for the number of calibration attempts for *eye-tracking condition* only.

| | (1) Dictator Game | (2) Trust Game (Player 1) | (3) Trust Game (Player 2) | (4) Holt and Laury | (5) Double Auction | (6) Eckel and Grossman | (7) Public Goods | (8) Ultimatum Game (Player 1) | (9) Ultimatum Game (Player 2) | (10) Cheating Game |
|---|---|---|---|---|---|---|---|---|---|---|
| Number of Calibration | -0.357 | -0.033 | 0.017 | 0.185*** | 0.050 | 0.220** | -0.823 | -0.048 | 0.000 | 0.063 |
| | (0.204) | (0.021) | (0.026) | (0.057) | (0.063) | (0.085) | (1.004) | (0.125) | (0.046) | (0.111) |
| Initial Offer | | | | | | | | | 0.067 | |
| | | | | | | | | | (0.045) | |
| Constant | 3.183*** | 0.474*** | 0.307*** | 4.300*** | 1.377*** | 4.116*** | 63.397*** | 4.873*** | 0.513 | 4.437*** |
| | (0.501) | (0.077) | (0.073) | (0.325) | (0.315) | (0.333) | (5.881) | (0.460) | (0.275) | (0.676) |
| N | 46 | 46 | 37 | 122 | 122 | 72 | 72 | 36 | 36 | 72 |
| R-squared | 0.032 | 0.041 | 0.016 | 0.035 | 0.006 | 0.047 | 0.003 | 0.003 | 0.062 | 0.006 |

Note. Coefficients from OLS estimation are reported. Standard errors are presented in parentheses and clustered at the session level. Since the *no eye-tracking condition* has no variation in calibration attempts, the estimates include only the *eye-tracking condition.* The dependent variables used in each game are: the number of tokens sent in Dictator game; the proportion of the number of tokens sent is used for Player 1, whereas the reciprocity is used for Player 2 in Trust game; the number of safe choices in Holt and Laury risk task; the average profits for 10 periods in Double Auction; the number of safe choices in Eckel and Grossman risk task; the average number of tokens kept in Public Goods game; the number of tokens sent is used for Player 1, whereas the acceptance rate is used for Player 2 in Ultimatum game; the average number of tokens reported for 10 periods in Cheating game. The results with controls are functionally equivalent and are available from the authors upon request. * Statistically significant at 10% level; ** at 5% level; *** at 1% level.

Table D7: Effect of difficulty calibrating on emotions as measured by face recognition software.

| | (1) Joy | (2) Anger | (3) Surprise | (4) Fear | (5) Contempt | (6) Disgust | (7) Sadness |
|---|---|---|---|---|---|---|---|
| | Panel I: Holt and Laury | | | | | | |
| Trouble Calibrating | 2.091 | -0.762 | 0.688 | 2.930* | -1.832 | -0.743 | -1.605 |
| | (2.530) | (1.779) | (2.337) | (1.687) | (2.475) | (0.867) | (3.016) |
| N | 101 | 101 | 101 | 101 | 101 | 101 | 101 |
| R-squared | 0.01 | 0.00 | 0.00 | 0.03 | 0.00 | 0.01 | 0.00 |
| | Panel II: Eckel and Grossman | | | | | | |
| Trouble Calibrating | 7.423*** | -0.275 | -2.662 | 1.840* | -1.911 | -0.083 | -0.203 |
| | (1.888) | (0.570) | (3.037) | (0.988) | (2.755) | (0.646) | (0.297) |
| N | 62 | 62 | 62 | 62 | 62 | 62 | 62 |
| R-squared | 0.205 | 0.00 | 0.01 | 0.06 | 0.01 | 0.00 | 0.01 |

Note. Each column represents the results from a separate OLS regression. Robust standard errors are in parentheses. Trouble calibrating is 1 if the subject is in the worst 10 percent of calibrators for each game. Number of observations are 101 in the first panel and 62 in the second panel. Observation numbers decrease compared to Table 1 because of lack of sensitivity in the facial recognition software. * Statistically significant at 10% level; ** at 5% level; *** at 1% level.

# References


[1] Engel C. Dictator Games: A Meta Study. Experimental Economics. 2011; 14(4): 583–610.

[2] Eckel CC, Wilson RK. Is Trust a Risky Decision?. Journal of Economic Behavior & Organization. 2004; 55(4): 447–465.

[3] Johnson ND, Mislin AA. Trust Games: A Meta-Analysis. Journal of Economic Psychology. 2011; 32(5): 865–889.

[4] Holt CA, Laury SK. Risk Aversion and Incentive Effects. The American Economic Review. 2002; 92(5): 1644–1655.

[5] Kassas B, Palma MA, Porter M. Happy to Take Some Risk: Investigating the Dependence of Risk Preferences on Mood Using Biometric Data. 2019.

[6] Neri, C. Eliciting Beliefs in Continuous-Choice Games: A Double Auction Experiment. Experimental Economics. 2015; 18(4): 569–608.

[7] Eckel CC, Grossman PJ. Sex Differences and Statistical Stereotyping in Attitudes Toward Financial Risk. Evolution and Human Behavior. 2002; 23(4): 281–295.

[8] Zelmer J. Linear Public Goods Experiments: A Meta-Analysis. Experimental Economics. 2003; 6(3): 299–310.

[9] Eckel CC, Harwell H. Four Classic Public Goods Experiments: A replication Study. In Replication in Experimental Economics. 2015. Emerald Group Publishing Limited.

[10] Oosterbeek H, Sloof R, Van De Kuilen G. Cultural Differences in Ultimatum Game Experiments: Evidence from a Meta-Analysis. Experimental Economics. 2004; 7(2): 171–188.

[11] Aksoy B, Palma MA. The Effects of Scarcity on Cheating and In-Group Favoritism. Journal of Economic Behavior & Organization. 2019; 165: 100–117.
