# Supplementary material for: Does eye-tracking have an effect on economic behavior?
Source: PLoS One. 2021 Aug 5;16(8):e0254867. doi: 10.1371/journal.pone.0254867 (PMC8341649; doi:10.1371/journal.pone.0254867)
Supplement: S1 Data — (ZIP) [file pone.0254867.s005.zip › Data/ReadMe.rtf]

**********************************************************************************************************************“Does eye-tracking have an effect on economic behavior?”: Replication Code**********************************************************************************************************************This ReadMe describes the folder structure to enable users to replicate the results of this paper. There are two folders: data and codes. Data folder has three data (main.dta, da.dta, sensor.dta) and the corresponding analyses are conducted using .do files (main.do, da.do, sensor.do) in codes folder.1. Data 1) main.dta contains a main data collected from Z-tree.2) da.dta has additional double auction data (bid, ask, transaction price, and transaction volume) collected from Z-tree.3) sensor.dta consists of main data from Z-tree and corresponding sensor data (eye-tracking and facial expression data).2. Codes1) main.do is a stata file that conducts main analyses in the manuscript and the supplemental materials using main.dta.2) da.do is a stata file that analyzes the additional double auction dimension using da.dta. 3) sensor.do is a stata file that provides the analyses for eye-tracking and facial expression in the manuscript and the supplemental materials using sensor.dta.
