# Supplementary material for: Does eye-tracking have an effect on economic behavior?
Source: PLoS One. 2021 Aug 5;16(8):e0254867. doi: 10.1371/journal.pone.0254867 (PMC8341649; doi:10.1371/journal.pone.0254867)
Supplement: S1 Data — (ZIP) [file pone.0254867.s005.zip › Data/data/Myfile.doc]

Descriptive Statistics 
 Variable	 Obs	 Mean	 Std. Dev.	 Min	 Max	
 male	71	.521	.503	0	1	
 age	72	21.861	4.997	0	39	
 edu	.	.	.	.	.	
 Freshman	72	.222	.419	0	1	
 Sophomore	72	.097	.298	0	1	
 Junior	72	.139	.348	0	1	
 Senior +	72	.208	.409	0	1	
 Master	72	.222	.419	0	1	
 Ph.D.	72	.111	.316	0	1	
 race	.	.	.	.	.	
 White	72	.431	.499	0	1	
 Black	72	.028	.165	0	1	
 Asian	72	.431	.499	0	1	
 Others	72	.111	.316	0	1	
 income	.	.	.	.	.	
 <$45k	72	.375	.488	0	1	
 $45k-$49k	72	.042	.201	0	1	
 $50k-$59k	72	.083	.278	0	1	
 >$60k	72	.5	.504	0	1	
	
