# Supplementary material for: Does eye-tracking have an effect on economic behavior?
Source: PLoS One. 2021 Aug 5;16(8):e0254867. doi: 10.1371/journal.pone.0254867 (PMC8341649; doi:10.1371/journal.pone.0254867)
Supplement: Attachment — Submitted filename: Response to Reviewers.pdf [file pone.0254867.s006.pdf]

Editor:

0. Revise the paper for a wider audience.

We have done our best to make the focus of the paper more applicable to a wider audience. Please see the changes particularly in the introduction, but also throughout the paper.

1. Please ensure that your manuscript meets PLOS ONE's style requirements, including those for file naming. The PLOS ONE style templates can be found at

https://journals.plos.org/plosone/s/file?id=wjVg/PLOSOne_formatting_sample_main_body.pdf and

https://journals.plos.org/plosone/s/file?id=ba62/PLOSOne_formatting_sample_title_authors_affiliations.pdf

Removed zip codes and postal codes from author affiliations.

Removed funding information and ethics statement from Acknowledgements.

Moved figures to separate files as .tif or .tiff extensions.

2.Please amend your current ethics statement to include the full name of the ethics committee/institutional review board(s) that approved your specific study.

Once you have amended this/these statement(s) in the Methods section of the manuscript, please add the same text to the “Ethics Statement” field of the submission form (via “Edit Submission”).

3. If you wish to make changes to your Data Availability statement, please describe these changes in your cover letter and we will update your Data Availability statement to reflect the information you provide.

We will do this once the paper is accepted.

4. Your ethics statement should only appear in the Methods section of your manuscript. If your ethics statement is written in any section besides the Methods, please move it to the Methods section and delete it from any other section.

The ethics statement has been moved to appear only in the Methods section.

Reviewer 1

1. A "non-invasive" and practically imperceptible eye-tracking technique should not cause any change in behavior.

*We 100% agree with this statement, especially now that our results have confirmed what we suspected prior to running the study. Eye-tracking **IS** non-invasive and should not cause a Hawthorne effect from social desirability bias.*

However, other people, particularly those who do not work in eye-tracking research, are very concerned about this possibility, which is why we were motivated to write this paper. We have received comments and referee reports that there could be a Hawthorne effect including grant proposals for eye-tracking research from reviewers (even at NSF!) and also in seminars and conferences. Most recently, one of the authors gave a seminar on a different eye-tracking project at Harvard School of Public Health and it was great to be able to tell the audience member who asked about it that we find no Hawthorne effect in this study even with a cheating game. That seemed to satisfy the audience member's concerns.

Other eye-tracking researchers have told us they also have to sometimes justify the lack of a Hawthorne effect on grant proposals and to reviewers, even though they also think any effects are unlikely, or if they exist, they are of short duration. The eye-tracking hardware company, Tobii, is also excited about our results and they asked us to present these results at a recent conference.

Because we expected a null result, we made sure to have more observations than power analysis called for so that we could defend a null result. We also pre-registered this experiment with the SDB effect results, but not with the unexpected calibration effect results.

We believe that addressing the issue of a potential eye-tracking effect on behavior in a systematic way will increase the visibility of the paper and this is why we chose PLOS ONE as our preferred outlet, because we believe this journal will reach a wide audience of social scientists and may be informative to researchers who are already using eye-tracking and encouraging for those who are contemplating its use.

We discuss concerns about experiments and behavioral modification more generally starting on page 2 (with the Schram and Charness (2015) and Barmettler, Fehr, and Zehnder (2016 citations), Lines 50-54 and also more specifically on page 2, Lines 83-85.

2. References on eye images and using glasses are irrelevant.

There was so little research in this area (possibly because of the null findings, which can be difficult to publish, even though their results are important), these were the closest references that we could find to what we were trying to do. It is possible that there may be publication bias against null results findings and this is why we were not able to find many relevant references, other than those that go out of their way to make eye-tracking salient. We agree that they are straw-men and not really relevant (other than that even their Hawthorne effects are short-lived) and have greatly shortened their descriptions in the introduction and on page 2, Lines 75 - 89.

3. More needs to be done to test the Calibration Effect, some of which is theoretically impossible.

   Right now, although the paper talks about eye-tracking effects, but the data and the statistical results are more about calibration effects.

We apologize for the lack of clarity-- this paper is meant to be about eye-tracking effects writ-large with a specific emphasis on Social Desirability Bias because it is the pathway that reviewers and panelists are most often worried about. The calibration effects are a subset of eye-tracking effects that we did not put into our pre-registration. We had expected that if there were any eye-tracking effects at all, we would find them in the cheating game and that it would go away after a few minutes or a few rounds.

The null effects results are the main results of the paper that we want to focus on. These are the results that we expect to be useful to eye-tracking researchers in grant proposals and refereed articles. That said, any deviation from null effects in a null effects paper must be explored. The fact that we found a potential effect in a risk preference game, which has low levels of SDB, led us to conduct our additional exploration into the calibration effects. Because we could not attribute that eye-tracking effect to SDB, we did further exploration, and found the suggestive results that could be explained by difficulty with calibration increasing risk aversion. Thankfully, even this eye-tracking should not be problematic for researchers doing eye-tracking because people who have had difficulty calibrating are often removed from the eye-tracking sample because they have poor eye-tracking data. Thus, the calibration effect should not be problematic for researchers and we find no other eye-tracking effects.

Because null effects results can be repetitive and boring to read about, we did our best to minimize the space they took up in the paper, which is why the games and figures are introduced in the same sections of their results rather than presenting them separately. It is much harder to condense the calibration results because they are not repetitive. Still, we have shortened the calibration results in this draft and signaled up

front that these calibration results are not the point of the paper (page 3, Lines 126-134), but still something worth investigating that eye-tracking researchers should be aware of and that requires future research with other risk aversion measures.

We also deleted the section on emotions and the former final table on biometric analysis. If the reviewers and editors would like, we are open to shortening the calibration results further, by, for example, moving some of the tables to appendices and just reporting the results in the paper.

We believe that another separate paper would need to be done to fully explore the calibration effect-- one that has been designed and pre-registered for such a purpose. Our current design is not ideal for testing this as it was not the main purpose of our work.

4. Report technical details of the device. Explain the calibration process and whether all observations were included in the statistical analysis or not. Control for all those details in the statistical analysis.

We have added information about the technical details of the device and the calibration process on page 4, Lines 156 - 183. All observations were included in the statistical analysis except those noted in S4 Appendix D that were lost in a computer failure, and of course those we purposefully dropped because of poor calibration or poor data quality in Table 6. We cannot control calibration attempts in this setup: eye-tracking details can only be controlled for the treatment group-- the control group has no variation in calibration attempts and the treatment group has at least one per game. No control group members have failed calibration attempts, so that is also collinear with the treatment. All other details were consistent across participants. Table D6 in S4 Appendix D demonstrates that the number of calibration attempts only significantly affects the two risk aversion games.

When controls are added, 2 observations drop in the Group 1 games because subjects did not provide their income level. Similarly, one subject in Group 2 did not report gender. In Table D1 S4 Appendix D in which probit results are reported for Player 2 in the Ultimatum game, 3 observations for sophomore, 1 observation for Black, 7 observations for Others,and 3 observations for $50,000-$59,000 were dropped because these observations predict success perfectly (the main table reports OLS results for ease of interpretation, but results are not appreciably different even with these drops). Note that the eye-tracking results for all games are very similar regardless of whether controls are included, so neither the observations dropping nor the controls are changing the eye-tracking effect.

5. When analyzing acceptance decisions from the ultimatum game, control for the size of the initial offer.

We swapped out the Table 3 results for the ultimatum game to results that include a control for size of the initial offer and moved the original results without this control to Table D1 in S4 Appendix D. The results for the eye-tracking variable are nearly identical.

6. Control for time trends in the public-goods game and the double auction.

We discuss these results on page 11, Line 409 and page 12, Line 467 and show these results in Table D4 in S4 Appendix D. The results for the eye-tracking variable are nearly identical.

7a. Remove the term "marginally significant" and refrain from interpreting "differences" with a p-value larger than 0.05.

We have removed these throughout the paper. Any remaining discussion of magnitudes with p-values larger than 0.05 is there when it biases us against having a null finding (ex. Differences that are not robust to controls, or that go away as we remove poor calibrators).

Note that we have removed suggestive results about emotions and calibration in what had been the section "Biometric analysis for subjects who had trouble with eye-tracking calibration" because those results only had marginal significance.

7b. As a robustness test, check the OLS findings with a regression model that is more appropriate for count data (Holt-and-Laury task, Eckel-and-Grossman task).

We discuss these results on page 9, Lines 342 - 350 and in table D3 in S4 Appendix D. In our situation, the negative binomial is identical to the Poisson, so we report Poisson results only.

Reviewer #2

1. Lines 29 to 33. The authors employed tasks that have several levels of socially desirable choices. I wonder whether they examined differences (for example, in effect size) between tasks with no socially preferred choice and those that have.

The task with no predicted SDB is the Auction game and there is no difference in effect size between the eye-tracking and non-eyetracking condition. The game with the highest predicted SDB is the cheating game, and again, there is no difference in effect

size between the eye-tracking and non-eyetracking conditions. So for the two extreme games, there is no difference in effect size. The actual magnitudes cannot be directly compared because the outcomes are different (price and quantity vs. average reported number), and normalizing them would make them equivalent to each other mechanically (mean 0, sd 1). Thank you for your comment as it is useful in our motivation. If we misunderstood, please let us know and we will do our best to respond.

2. Explore whether there is a short-lived SDB by splitting each game in half and running separate analyses separately on each half.

It is possible that a small short-lived SDB would not show up in the completed game results, thus looking at just the early results could capture SDB that disappeared later. As suggested, we have done a separate analysis for each half of the game for all games that had multiple periods. We analyzed the first 5 rounds separately from the second 5 rounds in all 10 round games. For the three multi-period games for which we found no eye-tracking effect, the Double Auction, the Public Goods Game, and the Cheating Game, we still find no eye-tracking effect when limiting to just the first five games

It is interesting that for HL, in which we did find an eye-tracking effect, that effect seems to be limited to the first half of the game. So the eye-tracking effect we find is fleeting and suggests that another possible avenue for researchers concerned about eye-tracking effects on risk aversion games is to only use the later data for analysis purposes. (Alternatively, since the rounds are sequential, it may be that the marginal effect of eye-tracking on risk aversion occurs within the first five rounds of the game.)

We discuss the results for HL in the abstract, and on page 3, beginning at 118, page 9, beginning at 328, page 13, beginning at Line 524, and page 16, Line 621 and we report these results in Table 4, Line 372/S2 Appendix B Figures B1-B4.

3. Lines 610 to 612. Differences in the number of calibration attempts across tasks should be included, even if they are not expected—also, an explanation of why calibration attempts affect the HL but not the others.

Because the calibration happens before each game, the game itself should not affect the calibration. However, the measure we are using is a cumulative calibration attempts measure. We have added a table with the number of calibration attempts as Table D5 in S4 Appendix D. We suspect that we would have seen a similar significant eye-tracking calibration effect with the EL risk-aversion game if there had been more failed calibration attempts prior to it.

4. Line 626. Could they explain a bit further how the estimation of the emotional states works?

Based on the comments from another reviewer and after very careful consideration, we have decided to delete the details of the discussion about emotions and how they may induce changes in risk. While we believe this is an interesting research topic, one of the reviewers indicated that adding this discussion may detract from the main research question of this paper about potential Hawthorne effects of using eye tracking and was also concerned about drawing conclusions from results that were not significant at the 5 percent level.

There is an emerging literature that links emotions and risk. We feel this can be a stand alone contribution of a separate paper. We are looking forward to investigating this question in future work in a more robust design better tailored to investigate this research question. Note that if the reviewers and editor disagree with this choice, we are open to reintroducing or expanding this topic to the paper.

5. Line 71-> Name the Hawthorne effect earlier when including the impact of being observed in other studies so that a reader that is not familiar with the term can fully understand its relevance (for example, between lines 39 to 45).

Done, we have moved this earlier.

6. Line 284 -> a “T” in theoretically

Fixed.

7. Line 287-> Frey and Bohnet find(without the final “s”)

Fixed.
